# Effect of Breast Screening Regimen on Breast Cancer Outcomes: A Modeling Study

**DOI:** 10.3390/curroncol30110686

**Published:** 2023-10-25

**Authors:** Martin J. Yaffe, James G. Mainprize

**Affiliations:** 1 Physical Sciences Platform, Sunnybrook Research Institute, Toronto, ON M4N 3M5, Canada; james.mainprize@sri.utoronto.ca; 2 Department of Medical Biophysics, University of Toronto, Toronto, ON M5G 1L7, Canada; 3 Ontario Institute for Cancer Research, Toronto, ON M5G 0A3, Canada

**Keywords:** breast cancer, screening, regimen, mortality, stage at detection, microsimulation modeling, number needed to screen

## Abstract

Guidelines vary for the age at which to begin breast cancer screening and the interval between examinations. A validated computer model was used to compare estimated outcomes between various screening regimens. The OncoSim-Breast microsimulation model (Canadian Partnership Against Cancer) was used to simulate a cohort of 1.53 million Canadian women born in 1975. The effect of screening regimen on absolute breast cancer mortality rates, stage at diagnosis, number needed to be screened to avert a breast cancer death or save a life year, abnormal recall rates and negative biopsy rates was examined for unscreened women or those entering screening at age 40 or 50 and screened annually or biennially to age 74. Compared to no screening, absolute mortality reduction was 4.6 (biennial 50–74), 5.9 (biennial 40–74) and 7.9 (annual 40–74) fewer deaths per 1000 women. The absolute rate of diagnosis of advanced cancers (Stage 2, 3 and 4) falls in favor of earlier stages as the number of lifetime screens increases. Annual screening beginning at age 40 until age 74 would provide an additional reduction of 2 and 3.3 breast cancer deaths per 1000 women compared to biennial screening beginning at ages 40 and 50, respectively. There is a corresponding drop in the absolute number of Stage 2, 3 and 4 cancers diagnosed.

## 1. Introduction

Breast screening with mammography has been demonstrated to contribute to the reduction in premature mortality from breast cancer [1,2]. This occurs by allowing the detection of the disease at an earlier point in its natural history. There continues to be considerable controversy regarding the appropriate ages at which women might begin attending screening and discontinue screening as well as the optimum interval for screening examinations to take place, and these have been reflected in differences in the guidance on screening [3,4,5]. 

In 2023, the US Preventive Services Task Force issued draft recommendations for screening suggesting that women facing average risks of breast cancer consider biennial screening between ages 40 and 74 years, reducing the previously recommended starting age of 50 years [6]. In order to gain insight into the possible implications of adopting this regimen, we updated previous calculations performed with an earlier breast cancer model, Model W, developed by The NCI-supported Cancer Intervention and Surveillance Modeling Network (CISNET) [7]. We employed a new validated microsimulation model, OncoSim-Breast, to estimate outcomes, including the number of breast cancer deaths, the effect on life years saved and the stage at diagnosis for a number of regimens that are currently utilized or are of interest. In addition, predictions were made of the number of screening examinations, the number of women required to be screened to avert a breast cancer or to save a life year otherwise lost to breast cancer, the number of women recalled after screening and the number of negative breast biopsies for different regimens.

## 2. Materials and Methods

OncoSim-Breast is a microsimulation model that simulates cancer initiation, progression and growth, detection, treatment and outcomes. Details of the model have been described by Yong et al. [8] The model was developed jointly by The Canadian Partnership Against Cancer and Statistics Canada and was inspired in part by the CISNET Wisconsin/Harvard Model [9]. OncoSim-Breast has been used in several previous studies to assist in interpreting cancer data [10,11,12] and is available from the Partnership for use in research [13]. The model produces “histories” of individuals, some of whom, at various time points in their lives, are predicted to have breast cancer initiated. Model results are updated at a yearly rate for each history. In the model, cancers are distributed among different subtypes and also have a distribution of growth rates. As they grow and progress over time, the model assigns a stage to each cancer related to its size and modeled characteristics. The model has been calibrated such that a predicted cancer incidence is in good agreement with empirical Canadian breast cancer data [4]. 

The treatment that is appropriate for the subtype and size of each diagnosed cancer and the age at diagnosis is also simulated by the model using survival data from the published literature. 

In OncoSim, if a cancer is present, in most cases, it will be detected at some point by the woman herself, in a clinical examination or by screening. Some cancers will not be detected before a woman dies from some other causes. This is most likely to be the case for slow-growing cancers and for cancers that develop later in life when competing causes of mortality are greater. The model follows women to death or age 109, whichever occurs first (although results are currently reported only to Calendar Year 2051). Canadian life table data for females are utilized to account for causes of death other than breast cancer as the cohort of individuals ages. In addition, this allows for life years to be tracked so that the loss of life years due to breast cancer or those saved by the patient’s recovery due to earlier detection and treatment can be estimated. 

The model can also be configured to create and analyze cohorts of women with different risk factors; however, for the work reported here, an average Canadian population was considered. The screening examination consisted of a two-view digital mammography of both breasts as is currently performed in Canadian provincial and territorial screening programs.

The model was run for 1.53 million histories (women) for the regimens shown in the first column of Table 1, including “no screening”. The number of breast cancers diagnosed, the stage at diagnosis and the number of breast cancer deaths were estimated. By comparing these data between different regimens, the absolute number of breast cancer deaths averted and the number of life years gained per 1000 women as well as the relative breast cancer mortality reduction and the number of breast cancers deaths that would be averted each year in Canada were computed. In addition, the model was used to estimate the mean lifetime number of screening examinations per woman and the number of women required to be screened per breast cancer death averted or per life year gained. Finally, because screening will ultimately lead to some women who do not have breast cancer being recalled for additional imaging examinations, a fraction of them receiving biopsy procedures that will turn out to be negative for cancer, estimates of the number of these events were also provided.

## 3. Results

Table 1 presents, for each screening regimen, the estimated number of breast cancer deaths (occurring up to age 76) per 1000 women at age 40, absolute reduction of deaths per 1000 women compared to no screening, relative breast cancer mortality reduction and the absolute number of breast cancer deaths that potentially could be averted annually in Canada. In addition, the number of life years gained over their lifetimes (compared to no screening) per 1000 women in the cohort is shown in the rightmost column.

**Figure 1:**
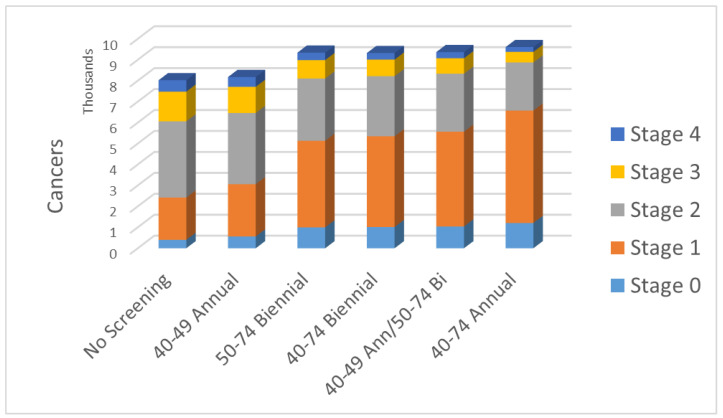
Absolute stage distribution of breast cancers diagnosed under different screening regimens.

Table 2 illustrates, for various screening regimens, the estimated absolute distribution of stages at diagnosis for breast cancers for a 36-year period of observation of an initial cohort of 100,000 40-year-old women. In this table, substages within Stages 1, 2 and 3 have been combined. The total number of cancers and the fraction that are diagnosed at early stages (1 or 2A) are also given. The effect of screening regimen on stage distribution is shown graphically in Figure 1.

Table 3 presents additional measures of performance of screening programs according to the regimen employed. These are given as the mean number of screens that each woman would receive over a lifetime, as well as the number of women required to be screened per breast cancer death averted and per life year gained. The number of recalls for imaging examinations for women without breast cancer and the number of biopsies which are negative for cancer in the cohort are also shown in this table.

## 4. Discussion

The OncoSim-Breast model predicts that attending a regimen of routine mammography screening will contribute to a marked reduction in the number of deaths from breast cancer. This is generally consistent with the results found by Coldman et al. in The Pan Canadian Study of Mammography Screening and Mortality from Breast Cancer [14], the observational studies conducted in European screening programs [15] and the CISNET model estimates conducted for the 2023 update by The US Preventive Services Task Force [16]. The model predicts that the absolute mortality reduction is greater when the age range is extended downward from ages 50 to 40 and also when screens occur annually versus biennially. Compared to the recommended regimen of biennial screening between ages 50 and 74 in place in many parts of Canada, annual screening from 40 to 74 is predicted to reduce the rate of breast cancer deaths by an absolute number of 3.3 per 1000 women (a 30% reduction), decreasing the number of breast cancer deaths in Canada each year by 788.

We can also consider the recent USPSTF recommendation of biennial screening between ages 40 and 74 [6]. Compared to biennial screening from 50 to 74, this would avert 1.4 deaths per 1000 women annually, providing slightly less than half the added benefit of the annual 40–74 regimen. Approaches to screening and treatment are somewhat similar in the US and Canada, so that the values shown in Table 1 are likely applicable to the US if the numbers in the second column from the right are increased by approximately 8.3, i.e., by the ratio of the populations of the two countries.

By comparing the two rightmost columns of Table 3, it is seen that when the reduction in life years lost to breast cancer is considered, the benefits of beginning screening earlier in life are more apparent. Considering the entries in the upper two rows of the table, the reduction of the starting age to 40 provides a 1.3-fold improvement in deaths averted but a 1.75 fold increase in life years gained by averting those deaths. Women who would develop and die of the disease at a younger age have more to gain from earlier detection than those whose disease occurs later. An estimate of the mean number of years gained by an individual’s avoidance of breast cancer death is given for each regimen by dividing the value in the second column from the right by that in the rightmost column. The mean saving is about 7.3 years for those screened biennially from age 50, 10 years for annual screening from age 40 and 13.7 years for women screened in their 40s. 

Our estimates differ markedly from those presented by The Canadian Task Force on Preventive Health Care (CTPHC) on the number of women required to be screened to avert a breast cancer death [17]. For women aged 40–49, the CTFPHC provides the number 1724 (although this appears to be for only 7 years of screening) while the number 306 provided by OncoSim-Breast points to an efficiency that is over 5 times higher. The CTFPHC 1000-woman diagram for that age group suggests that in screening those women, 1 breast cancer death would be averted, while OncoSim indicates that the number is 3.3 [18]. There is a similar discrepancy for older women, for which the Task Force estimates that, depending on age group, 1087–1300 women would have to be screened to avert a death, while OncoSim-Breast suggests that the number is approximately 220.

The cause for the discrepancy is not completely clear, but two factors may be important. First, the CTFPHC considered the number of women required to be *invited* into a trial of screening rather than those actually screened, and of course, the benefit of screening will only accrue to those who participate. Secondly, the CTFPHC based its assessment of benefits on old randomized trials conducted in an environment where both screening techniques and available therapies were less effective than those used today. 

In addition to the effect on mortality, one can consider the influence on stage distribution and the absolute number of cancers at each stage as illustrated in Table 2 and Figure 1. The effect of screening is to detect cancers at an earlier point in their natural history. Screening results in a marked increase in the detection of in situ cancers: some are destined to progress and some are not. Similarly, there is an increase in the number of cancers diagnosed at Stage 1, with the effect being monotonically greater as the intensity of screening is increased (beginning at 40 rather than 50 and annual rather than biennial). At the same time, both the absolute number and proportion of Stage 2, 3 and 4 cancers fall progressively as the lifetime number of screens in the regimen increases.

It is observed that the estimated total number of cancers diagnosed increases progressively from the “no screening” regimen, being 19% higher for annual screening between ages 40 and 74. This increase could be interpreted as due to the detection of cancers that progressed sufficiently slowly, which, in the absence of screening, would not have surfaced during the observation period. This is known as overdetection. We do not have sufficient knowledge of the internal workings of the OncoSim model to make this specific inference; however, part of the excess would certainly be expected to be due to the detection of nonprogressive Stage 0 (in situ) cancers. 

Overdetection of cancer becomes a harm if it leads to overtreatment of the disease. Once cancers are detected, it is their pathologic characterization that guides the choice and aggressiveness of therapy. The careful assessment of pathologic information including the judicious use of prognostic and predictive biomarkers can be useful in minimizing the occurrence of overdiagnosis and overtreatment. The large variability in published estimates of the amount of overdetection that occurs with screening currently makes it difficult to weigh its potential harms against the benefits of the reduction of mortality and morbidity associated with screening. At the same time, it should be noted that with the more intensive screening regimens, compared to no screening the absolute number of Stage 3 and Stage 4 cancers has dropped by 12.27 per 1000 women screened, a relative reduction of 62%.

Our findings are consistent overall with those reported in publications that utilized the CISNET models to examine the potential impact of different screening regimens [7,19]. 

As seen in Table 3, the benefits of more intensive screening described here are accompanied by increases in the number of recalls for further imaging after screening, most of which will ultimately be negative for the presence of cancer, as well as an increase in the number of negative biopsies. Of course, there is also an increase in the total number of screening examinations that must be performed. This has implication on costs and the resources required to conduct the screening; however, as suggested in the recent work of Wilkinson et al., a substantial fraction of these costs could be offset by a reduction of the cost of treating Stage 3 and 4 cancers through earlier detection [20]. 

The strengths of this study are the use of a dedicated Canadian model that has been validated against empirical data and provides flexibility in simulating different schedules for screening and the presentation of model predictions of stages at diagnoses [4,7,8]. Estimates of mortality reduction have been presented versus no screening in an absolute form in two ways: as the decrease in the number of breast cancer deaths or in life years saved per year per 1000 women in a cohort and for the female population of Canada (scalable to the US). From these values, the outcomes of different screening regimens can be compared by simple subtraction. In addition, relative mortality reduction has been presented for each regimen. The OncoSim-Breast model is available free from The Canadian Partnership Against Cancer to approved users for application to specific problems [13]. 

The model also has the provision of calculating costs of screening and therapy for cost-effectiveness studies; however, as indicated by Wilkinson et al., the treatment costs currently used in the model are now out of date and require revision to reflect current practice [20]. 

There are also limitations. The estimates provided here describe outcomes for 100% participation of cohort members with the screening regimen, i.e., that all women receive their examinations at the indicated protocol. Clearly, only those who actually participate in screening will derive its benefits, so the estimates represent the attainable effectiveness for participants. For a real-world cohort, the actual effectiveness will depend on the participation rate.

For any particular participation rate less than 100%, outcomes can be estimated from the data in Table 2 and Table 3 by a weighted combination of outputs based on model runs for multiple regimens. As an example, we can consider a recommended regimen of annual screening between ages 40 and 74, where the participation rate is 70%. In this case, a 70:30 weighted mixture of outcomes for annual screening from 40 to 74 years and no screening is calculated. From Table 1, the modified breast cancer death reduction is (7.9 × 0.7 + 0 × 0.3) = 5.5 per 1000 women in the cohort *offered* screening. Similarly, the number of premature breast cancer deaths averted each year in Canada would become 1862 × 0.7 = 1303, and the relative mortality reduction would fall from 50.4% to 35.3%; however, the number of life years saved per 1000 women (actually) screened would not change. At the same time, as seen in Table 3, reduced participation of 70% would result in a reduction by 30% in the total number of screens that would be performed across the cohort, the number of women who would be recalled after a suspicious screening exam and the number of negative biopsies. Once again, the number of women required to actually be screened to avert a breast cancer death or save a life year is not affected. Similar calculations can be performed for partial participation in the other regimens. 

For a screening program to be maximally effective, participation must be high. Participation is driven by women’s values and perceptions regarding the benefits and drawbacks associated with screening, and these, in part, will be influenced by access to information about screening. While there is ample scientific information in the peer-reviewed literature on the ability to reduce premature deaths and morbidity through earlier detection, participation is challenged by the high degree of polarization about screening in media articles, variability in the quality of some of the studies and in the positions taken by some health professionals regarding preventive interventions such as screening.

It is generally accepted that the decision to participate in screening or not should be that for a woman herself to make and the decision should be facilitated by accurate and accessible information from a primary health care provider or other means. If a decision to be screened is made, the woman should not be hindered from ready access to an examination, preferably in an organized program. 

There is a dire shortage of high-quality studies investigating the utility that women ascribe to the benefits of screening versus the disutility associated with screening recalls, negative biopsies and overdetection (detecting cancers through screening that otherwise would not be found before individuals die due to some other cause). The availability of better data on utilities would be invaluable for informing women’s personal decisions regarding participating in screening. 

Another limitation is that OncoSim-Breast is currently configured only to allow reporting of outcomes up to the year 2051. This results in 36 years of follow-up from entry of information for women screened in their 40s (between 2015 and 2024), while in the follow-up of those screened up to age 74 (final screen in 2049), deaths occurring after age 76 are not reported. This only has minimal effect on estimates of relative mortality reduction but, because of the restricted follow-up, does produce an underestimation of absolute risk reduction for all regimens except 40–49.

We can estimate the effect of this under-reporting by referring to data produced using CISNET Model W. In the CISNET Consortium, six independent academic teams collaboratively built separate models to describe the natural history of breast cancer in terms of processes: its growth, detection, treatment and outcomes [9,19]. Our earlier work preceded OncoSim-Breast and was based on the use of the CISNET Wisconsin/Harvard microsimulation model (Model W) modified to reflect Canadian breast cancer incidences [7,21]. Model W is the closest of the CISNET models to the algorithmic logic of OncoSim-Breast. In its microsimulation, all of the above processes are broken down into a series of events, each assigned a probability, obtained from empirical data describing breast cancer, screening and treatment. Person histories are created to follow an individual through her life with a selection of random numbers for each possible event, weighted by the event probabilities to determine the occurrence of initiation of a cancer and assign growth rate, subtype, etc.

Model W allows for reporting on more extended follow-ups, and to examine the effect of the restricted follow-up in OncoSim, we compared calculations with estimates previously obtained from the modified CISNET Model W. The comparison suggests that with follow-up to age 89, absolute mortality reduction in Table 1 would be greater by 2 deaths per 1000 for annual screening and 1.5 deaths/1000 for biennial screening from ages 40 to 74 [7]. We are currently working with the OncoSim developers to extend the reporting period. 

## 5. Conclusions

Benefits of routine mammography screening in terms of reduction of breast cancer deaths and additional life years saved are increased by beginning screening at age 40 and screening at annual rather than longer intervals. Specifically, annual rather than biennial screening of women from ages 40 to 74 could provide an additional reduction of approximately two breast cancer deaths (or 20.8 life years gained) per 1000 women each year. More intensive screening results in a downward shift in the absolute (as well as the relative) number of Stage 2, 3 and 4 cancers diagnosed in favor of earlier-stage cancers. We have also provided outcome estimates that, combined with appropriate utility data, can be used to weigh these benefits against the cost of performing more screens and the increased number of callbacks and negative biopsies that result from more intensive screening.

## Figures and Tables

**Table 1:** Number of breast cancer deaths per 1000 women alive at age 40, absolute reduction in deaths per 1000 women compared to no screening, relative breast cancer mortality reduction and the absolute number of breast cancer deaths that potentially could be averted annually in Canada for each regimen compared to no screening are shown for various screening regimens. Also shown are life years saved per 1000 women screened.

Regimen	Breast Cancer Deaths/1000 Women *	Reduction in Deaths/1000 Women **	Rel. Breast Cancer Mortality Reduction	Annual Reduction in Breast Cancer Deaths, Canada ***	LY Saved/1000 Women Screened **
No screening	15.7	-	-	-	-
50–74 biennial	11.1	4.6	29.10%	1074	33.2
40–74 biennial	9.7	5.9	37.80%	1397	58.1
40–49 ann., 50–74 bi	9.1	6.5	41.70%	1538	68.3
40–74 annual	7.8	7.9	50.40%	1862	78.9
40–49 annual	12.4	3.3	20.80%	770	44.8

* Evaluated from ages 40 to 76 (calendar year 2051), ** compared to no screening, *** compared to no screening, based on 235,820 women turning 40 each year. LY—life years. Except where noted, all data are shown as absolute values.

**Table 2:** Distribution of stage at diagnosis of breast cancers versus screening regimen. Absolute numbers of cancers for a cohort initially consisting of 100,000 women at age 40 are shown. Total number of cancers for each regimen and fraction of invasive cancers that are Stage 1 or 2a (early invasive) are also given. Standard error of the mean on total cancers values is approximately 450.

	No Screening	40–49 Annual	50–74 Biennial	40–74 Biennial	40–49 Ann/50–74 Bi	40–74 Annual
Stage 0	403	565	998	1015	1042	1208
Stage 1	2019	2496	4133	4334	4526	5367
Stage 2	3641	3400	2977	2869	2768	2299
Stage 3	1417	1246	878	790	737	503
Stage 4	545	469	354	314	294	231
Total cancers	8025	8176	9341	9322	9367	9608
Fract. early inv.	0.54	0.59	0.72	0.74	0.76	0.82

**Table 3:** For each screening regimen shown in the first column, the mean number of lifetime screens that each woman would receive, the number of recalls for additional imaging after suspicious screens and the number of negative breast biopsies that would be performed per 1000 women over their lifetimes are given. Efficiency of each screening regimen is also described in terms of how many women must be screened per breast cancer death averted or per additional life year gained by not dying of breast cancer.

Regimen	Lifetime No. of Screens/Woman	No. Recalls, No Cancer/1000 Women	No. Neg Biopsies/1000 Women Screened	No. Women That Must Be Screened to Avert One Death	No. Women That Must Be Screened/ Life Year Gained
No screening	-	-	-	-	-
50–74 biennial	11.6	641	57.1	219.5	30.2
40–74 biennial	16.3	959	85.3	168.9	17.2
40–49 ann., 50–74 bi	20.9	1236	110.0	153.4	14.6
40–74 annual	31.6	1772	157.7	126.7	12.7
40–49 annual	9.3	660	58.8	306.4	22.3

## Data Availability

The authors would be pleased to share data from model runs with other researchers upon reasonable request.
